# Associations of AMP and adenosine induced dyspnea sensation to large and small airways dysfunction in asthma

**DOI:** 10.1186/s12890-019-0783-0

**Published:** 2019-01-28

**Authors:** Claire A. Cox, Ilse M. Boudewijn, Sebastiaan J. Vroegop, Siebrig Schokker, Anne J. Lexmond, Henderik W. Frijlink, Paul Hagedoorn, Judith M. Vonk, Martijn P. Farenhorst, Nick H. T. ten Hacken, Huib A. M. Kerstjens, Maarten van den Berge

**Affiliations:** 1 Department of Pulmonary Diseases, University of Groningen, University Medical Center Groningen, PO box 30.0001, 9700 RB Groningen, The Netherlands; 2 Groningen Research Institute for Asthma and COPD, University of Groningen, University Medical Center Groningen, PO box 30.0001, 9700 RB Groningen, The Netherlands; 3 Department of Pulmonary Diseases, Martini Hospital Groningen, PO box 30, 033 9700 RM Groningen, The Netherlands; 4 Department of Pharmaceutical Technology and Biopharmacy, University of Groningen, Antonius Deusinglaan 1, 9713 AV Groningen, The Netherlands; 5 Department of Epidemiology, University of Groningen, University Medical Center Groningen, PO box 30, 001 9700 RB Groningen, The Netherlands

**Keywords:** Borg score, Dry powder adenosine, AMP, Provocation, Dyspnea

## Abstract

**Background:**

Bronchial provocation is often used to confirm asthma. Dyspnea sensation, however, associates poorly with the evoked drop in FEV_1_. Provocation tests only use the large airways parameter FEV_1_, although dyspnea is associated with both large- and small airways dysfunction. Aim of this study was to explore if adenosine 5′-monophosphate (AMP) and adenosine evoke an equal dyspnea sensation and if dyspnea associates better with large or small airways dysfunction.

**Methods:**

We targeted large airways with AMP and small airways with dry powder adenosine in 59 asthmatic (ex)-smokers with ≥5 packyears, 14 ± 7 days apart. All subjects performed spirometry, impulse oscillometry (IOS), and Borg dyspnea score. In 36 subjects multiple breath nitrogen washout (MBNW) was additionally performed. We analyzed the association of the change (Δ) in Borg score with the change in large and small airways parameters, using univariate and multivariate linear regression analyses. MBNW was analyzed separately.

**Results:**

Provocation with AMP and adenosine evoked similar levels of dyspnea. ΔFEV_1_ was not significantly associated with ΔBorg after either AMP or adenosine provocation, in both univariate and multivariate analyses. In multivariate linear regression, a decrease in FEF_25–75_ during adenosine provocation was independently associated with an increase in Borg. In the multivariate analyses for AMP provocation, no significant associations were found between ΔBorg and any large or small airways parameters.

**Conclusion:**

AMP and adenosine induce equally severe dyspnea sensations. Our results suggest that dyspnea induced with dry powder adenosine is related to small airways involvement, while neither large nor small airways dysfunction was associated with AMP-induced dyspnea.

**Trail registration:**

NCT01741285 at www.clinicaltrials.gov, first registered Dec 4th, 2012.

**Electronic supplementary material:**

The online version of this article (10.1186/s12890-019-0783-0) contains supplementary material, which is available to authorized users.

## Background

Airway hyperresponsiveness (AHR) is a distinct asthma characteristic. Bronchial provocation tests can be used to assess AHR, can help to diagnose asthma and monitor asthma control [1]. However, the patient’s dyspnea perception associates poorly with the provocation test [2]. In clinical practice, patients often experience dyspnea before the provocative agent causes the forced expiratory volume in the first second (FEV_1_) to drop 20% [3]. On the other hand, others experience no dyspnea even when the FEV_1_ has dropped more than 20% [4]. A provocation test is based on the FEV_1_, which is believed to be a marker for the larger airway [5]. However, dyspnea sensation is associated with both large- and small airways dysfunction [6–8]. To evaluate the small airways, for example, the forced expiratory flow between 25 and 75% of the expiration (FEF_25–75_) or the difference in resistance between 5 Hz and 20 Hz (R_5_-R_20_) measured with impulse oscillometry (IOS) can be used [5]. Provocation tests with subsequent IOS measurements have suggested that dyspnea induced with a provocative agent corresponds better to small- than to large airways dysfunction [3, 9, 10].

Provocation tests can be performed with either direct or indirect acting agents. Direct stimuli, such as histamine and methacholine, stimulate the airway smooth muscle, resulting in airway contraction [11]. Indirect stimuli, on the other hand, induce the release of mediators from inflammatory cells, such as histamine, leukotrienes, and prostaglandins causing airway contraction [12]. Examples of indirect stimuli are mannitol, nebulized adenosine 5′-monophosphate (AMP), and dry powder adenosine. The well-established AMP is dose restricted (as AMP becomes insoluble above 320–400 mg/mL) [13], whereas mannitol and the newly available dry powder adenosine are not [14]. AMP and dry powder adenosine are well tolerated by patients [15], but mannitol evokes discomforting cough [16, 17]. AMP and dry powder adenosine appear to act via the same indirect pathways, but can consist of differently sized particles. Nebulized AMP commonly has a mass median aerodynamic diameter (MMAD) between 5.1–8.5 μm [18], depending on the nebulizer settings and AMP concentration [18, 19]. Dry powder adenosine, on the other hand, can be produced with an MMAD as small as 2.6–2.9 μm [20], with a much smaller distribution in particle size which is independent of the dose [20]. Therefore, dry powder adenosine was postulated to reach the small peripheral airways to a larger extent compared to nebulized AMP, especially when inhaled at a low flow [21]. Thus, to target the small airways specifically, without a dose restriction and cough, adenosine may be valuable.

In this study we evaluated whether there is a difference between the perception of dyspnea induced with the assumed small airways trigger dry powder adenosine or the assumed larger airways trigger nebulized AMP. In addition, we evaluated for both triggers if the perception of dyspnea during a provocation test is more closely associated to changes in large- or small airways function.

## Methods

### Study design and patients

This study was performed with baseline data from the previously published OLiVIA study (clinical trial number: NCT01741285, www.clinicaltrials.gov) [22]. Included subjects were asthmatics (doctor’s diagnosis), current or ex-smokers (> 5 pack years), aged between 18 and 65 years, and all had a preserved lung function (FEV_1_ > 50%predicted and > 1.2 L). Excluded were subjects with a recent (< 6 weeks) exacerbation or upper airway infection, females who were pregnant or lactating, and subjects with clinically unstable concomitant diseases. The screenings phase of the OLiVIA study incorporated two provocation tests. First an AMP provocation and 14 ± 7 days later a dry powder adenosine provocation, performed after a washout period of four to six weeks for asthma maintenance therapy and eight hours for short acting β2-antagonists (SABAs). In the Olivia study only subjects with hyperresponsiveness to adenosine (≥20% drop in FEV_1_ on < 20 mg adenosine) were included. In the current study, all subjects who performed both provocation tests were accepted, on condition that they experienced dyspnea (increase in Borg > 1) evoked by the challenge.

### Measurements

#### Provocation tests

Wet nebulized AMP (MMAD 5.1–8.5 μm) [18] was administered in doubling concentrations ranging from 0.04 to 320 mg/mL. The AMP solutions were inhaled during two minutes of tidal breathing, without a breath-holding period, using the APS Pro System (CareFusion) with the SideStream nebulizer (Philips Respironics) at an output rate of 0.13 mL/min. Consecutive concentrations were inhaled at five-minute intervals until the concentration caused the FEV_1_ to drop ≥20% (PC_20_) or the highest concentration was administered.

Dry powder adenosine (MMAD 2.6–2.9 μm) [20] was administered in doubling doses of 0.04 to 80 mg. The powder was inhaled from functional residual capacity (FRC) to total lung capacity (TLC) at a low flow rate of 20 30 L/min guided by an inspiratory flow meter, as described previously [23]. After each inhalation subjects held their breath for 10 s at TLC to allow for optimal airway deposition [24]. The procedure was repeated at three-minute intervals until the administered dose evoked a ≥ 20% drop in FEV_1_ (PD_20_) or the highest dose was administered.

#### Pulmonary function tests

Before and after each provocation test pulmonary function tests were performed. In all subjects spirometry and IOS measurements were performed to obtain parameters for large (i.e. FEV_1_, R_20_) and small airways (i.e. FEF_25–75_, R_5_-R_20_), using the classification from the review by Van der Wiel et al. [5]. Due to availability of the measurement device, multiple breath nitrogen washout (MBNW) was only measured in a subset of subjects in one of the centers. MBNW provided the index for the ventilation heterogeneity of the acinar (S_acin_) and conductive airways (S_cond_), and the lung clearance index (LCI).

#### Dyspnea score

Before and after the provocation test dyspnea was assessed with the Borg dyspnea score [25], scoring dyspnea sensation from 0 = ‘no dyspnea at all’ to 10 = ‘maximal dyspnea’.

### Statistical analysis

All analyses were performed on the change (Δ) in a parameter induced by the provocation test; calculated by subtracting the pre-provocation value from the post-provocation value. To check if adenosine and AMP induced similar responses, we compared changes in parameters between the two tests with a two sided Student’s paired t-test or a two-sided Wilcoxon test, in accordance with the normality of distribution. With Spearman’s correlation the change in Borg score (ΔBorg) was univariately correlated to the change in each parameter of spirometry, IOS and MBNW, for both AMP and adenosine. Subsequently, multivariate linear regression models were constructed, to investigate the origin of dyspnea. A large- and a small airways parameter from both spirometry and IOS, was selected for the model. The parameter had to have the lowest *p*-value in the univariate correlation analysis and were corrected for co-linearity (correlation < 0.7). Because of assumed clinical relevance, gender and smoking status were added to the model. Models were ran once without reducing or increasing the amount of parameters. As MBNW was measured in fewer subjects, a separate model was constructed expanding the models with the MBNW parameter with the lowest *p*-value.

## Results

### Study population

For this study 77 subjects were screened. However, 18 subjects were excluded as they were unable to perform spirometry or a provocation test adequately (*n* = 5), complete the medication washout period (*n* = 8), had chronic non-asthmatic respiratory diseases (*n* = 2), or had other unstable non-respiratory diseases (*n* = 3) [22]. A total of 59 subjects underwent both provocation tests of which 36 performed a MBNW test. Baseline characteristics are shown in Table 1.

**Table 1:** Baseline characteristics

Gender (M/F)	24/35
Age (years)	47.0 (37.0–55.0)
BMI (kg/m^2^)	26.8 (23.1–31.4)
Smoking status (Current/Ex)	30/29
Number of packyears (years)	16.8 (11.0–26.0)
Adenosine provocation (pos/neg)	45/14
Positive Adenosine (mg)	3.11 (0.87–6.38)
AMP provocation (pos/neg)	40/19
Positive AMP (mg/mL)	14.67 (4.7–44.88)
Borg score (points)	0.0 (0.0–2.0)
FEV_1_ (L)	2.93 (2.36–3.44)
FEV_1_ percentage of predicted (%)	85 (74–96)
FVC (L)	4.14 (3.52–4.94)
FVC percentage of predicted (%)	105 (94–116)
FEV_1_/FVC (%)	70 (62–77)
FEF_25_	4.86 (3.46–6.42)
FEF_25_ percentage of predicted (%)	72 (48–96)
FEF_50_	2.35 (1.70–3.27)
FEF_50_ percentage of predicted (%)	51 (36–65)
FEF_75_	0.67 (0.46–1.14)
FEF_75_ percentage of predicted (%)	36 (25–56)
FEF_25–75_	1.79 (1.30–2.74)
FEF_25–75_ percentage of predicted (%)	49 (35–65)
R_5_ (kPa sL^− 1^)	0.53 (0.42–0.67)
R_20_ (kPa sL^−1^)	0.42 (0.35–0.47)
R_5_-R_20_ (kPa sL^− 1^)	0.08 (0.04–0.22)
AX (kPa L^− 1^)	0.64 (0.24–1.82)
X_5_ (kPa sL^− 1^)	−0.13 (− 0.22- -0.09)
F_res_ (s^− 1^)	16.78 (12.33–21.83)
LCI_2.5%_^a^	9.27 (8.60–11.28)
LCI_5%_^a^	6.22 (5.76–7.37)
S_cond_^a^	0.04 (0.02–0.06)
S_acin_^a^	0.14 (0.10–0.19)

Data is presented as count or median (inter quartile range (IQR)). *pos* positive response, ≤ 20 mg for adenosine and ≤ 160 mg/ml for AMP; *neg* negative response, > 20 mg for adenosine and > 160 mg/ml for AMP, *FEV*_*1*_ forced expiratory volume in the first second, *FVC* forced vital capacity, *FEF*_*25*_ forced expiratory flow at 25% of FVC, *FEF*_*50*_ forced expiratory flow at 50% of FVC, *FEF*_*75*_ forced expiratory flow at 75% of FVC, *FEF*_*25–75*_ forced expiratory flow at 25 to 75% of FVC, *R*_*5*_ resistance to 5 Hz, *R*_*20*_ resistance to 20 Hz, *R*_*5*_*-R*_*20*_ difference in resistance to 5 Hz and 20 Hz, *AX* reactance area, *X*_*5*_ reactance to 5 Hz, *F*_*res*_ resonance frequency, *LCI* lung clearance index, *S*_*cons*_ ventilation heterogeneity of the conducting airways, *S*_*acin*_ ventilation heterogeneity of the acinar airways. ^a^ = multiple breath nitrogen washout (MBNW) was measured in 36 subjects

### Comparison of adenosine and AMP provocation

**Fig. 1:**
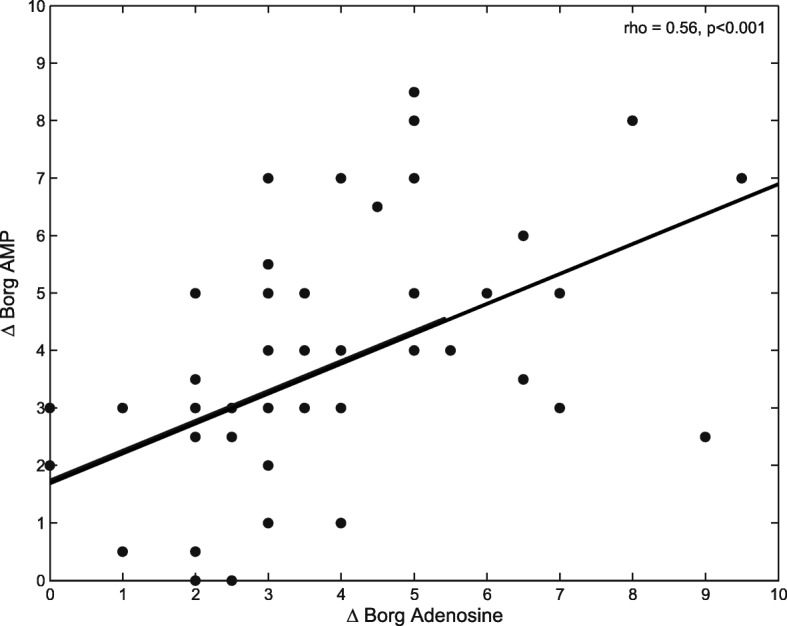
Scatter plot of correlation between change(Δ) in Borg Adenosine and Borg AMP

**Fig. 2:**
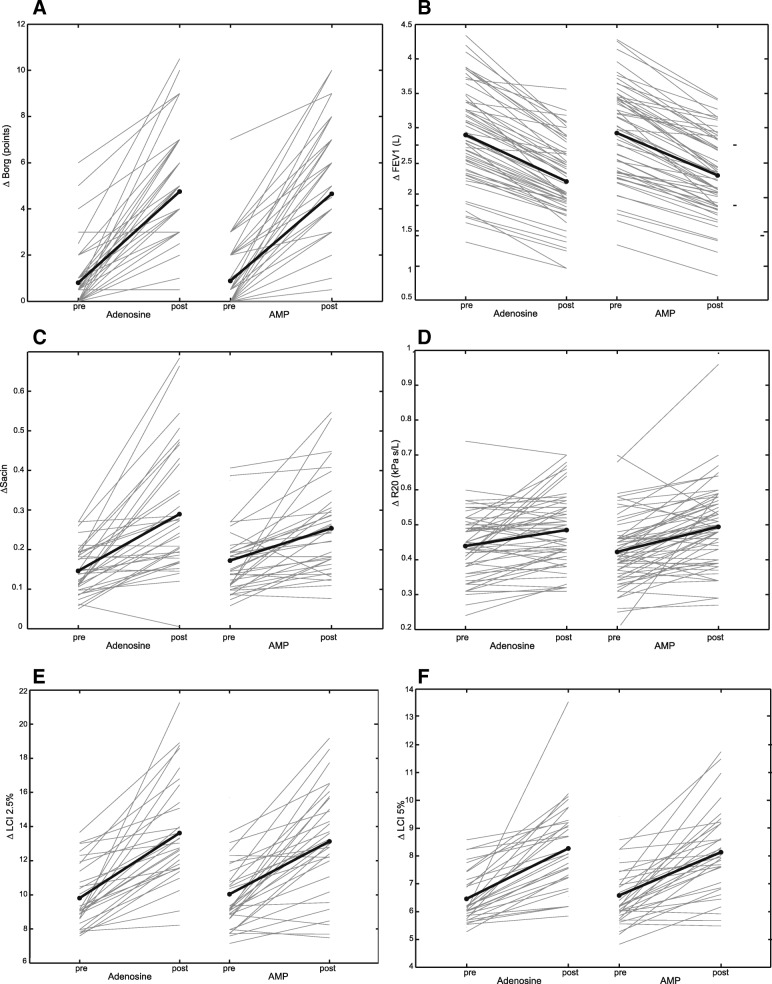
Comparison of dry powder adenosine to AMP provocation. **a**. the change in Borg dyspnea score (ΔBorg), **b**. the change in the forced expiratory volume in 1 s (ΔFEV_1_), **c**. the change in the resistance of the respiratory system to 20 Hz (ΔR_20_), **d**. the change in lung clearance index reaching 2.5% of the starting nitrogen concentration in the lung (ΔLCI_2.5%_), **e**. the change in the lung clearance index reaching 5% of the starting nitrogen concentration in the lung (ΔLCI_5%_), **f**. the change in ventilation heterogeneity of the accinar airways (ΔS_acin_)

Provocation with adenosine and AMP evoked a decreases in FEV_1_ of 23.4 ± 8% and 21.1 ± 8%, respectively. The severity of dyspnea evoked with adenosine and AMP was not significantly different, with an increase in Borg of 3.95 ± 2.1 and 3.77 ± 2.1 points, respectively (*p* = 0.65). Spearman’s correlation between ΔBorg after adenosine and ΔBorg after AMP was moderate (rho 0.56, *p* < 0.001) (Fig. 1). AMP provocation evoked a greater increase in R_20_ (*p* = 0.04) compared to adenosine, while adenosine evoked a greater increase in LCI_2.5%_ (*p* = 0.03) and S_acin_ (*p* = 0.01) (Fig. 2). An overview of all comparisons is shown in (see Additional file 1: Table S1).

### Univariate associations with ΔBorg

**Fig. 3:**
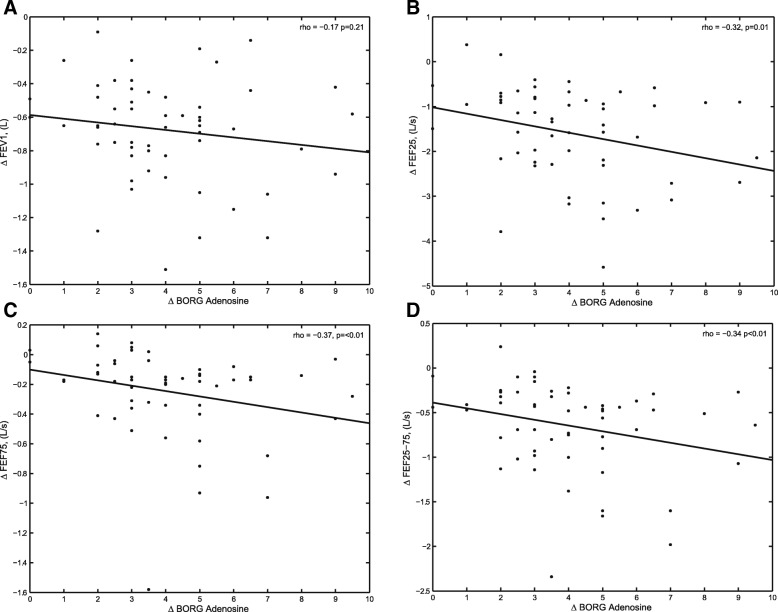
Dry powder adenosine: Spearman’s correlations to change in Borg dyspnea sensation (ΔBorg). **a**. the change in the forced expiratory volume in 1 s (ΔFEV_1_), **b**. the change in forced expiratory flow at 25% of forced vital capacity (FVC) (ΔFEF_25_), **c**. the change in forced expiratory flow at 75% of FVC (ΔFEF_75_), and **d**. the change in forced expiratory flow at 25 to 75% of the FVC (ΔFEF_25–75_)

**Fig. 4:**
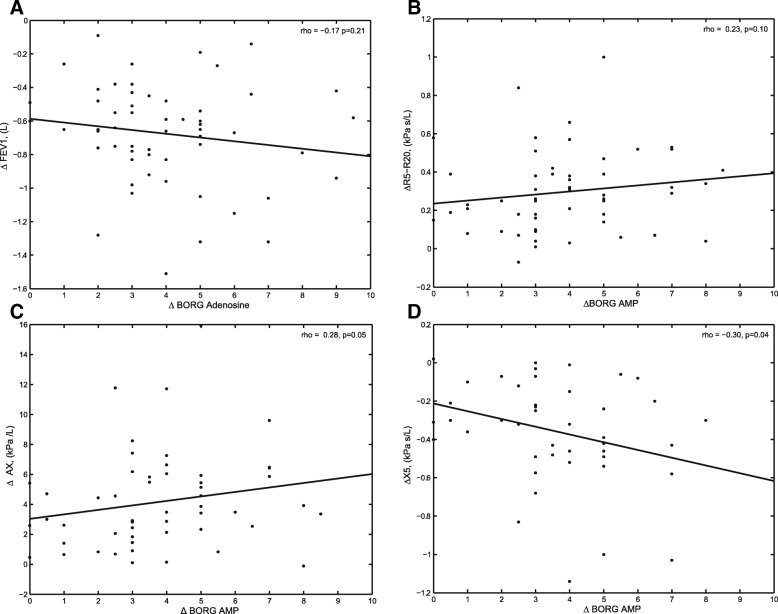
AMP: Spearman’s correlations to change in Borg dyspnea sensation (ΔBorg). **a**. the change in forced expiratory volume in 1 s (ΔFEV_1_), **b**. the change in the difference in resistance of the respiratory system to 5 Hz and 20 Hz (ΔR_5_-R_20_), **c**. the change in reactance of the respiratory system to 5 Hz (ΔX_5_), and **d**. the change in reactance area (ΔAX)

In the univariate analyses, ΔBorg for provocation with adenosine was significantly correlated with ΔFEF_25_ (Ls^− 1^), ΔFEF_75_ (Ls^− 1^), and ΔFEF_25–75_ (Ls^− 1^) and showed a trend toward an association with the ΔFEF_50_ (Ls^− 1^) (Fig. 3). The ΔBorg for provocation with AMP was significantly associated with ΔAX (kPa L^− 1^) and ΔX_5_ (kPa sL^− 1^) and there was a trend towards a correlation with ΔFEV_1_ (L) and ΔR_5_-R_20_ (kPa sL^− 1^) (Fig. 4). Results of all correlation analyses are shown in (see Additional file 1: Table S2).

### Multivariate associations with ΔBorg

#### Spirometry-IOS models

The multivariate model for adenosine included ΔFEV_1_ (L), ΔFEF_25–75_ (Ls^− 1^), ΔR_20_ (kPa sL^− 1^) and ΔR_5_-R_20_ (kPa sL^− 1^) (Table 2). This model showed an independent significant, negative association of ΔFEF_25–75_ (Ls^− 1^) with ΔBorg (R^2^ = 20.9%). The model for AMP included ΔFEV_1_ (L), ΔFEF_50_ (Ls^− 1^), ΔR_20_ (kPa sL^− 1^), and ΔX_5_ (kPa sL^− 1^) (Table 2) and showed no independent associations to ΔBorg (R^2^ = 4.3%).

**Table 2:** Multivariate models predicting ΔBorg in AMP and adenosine provocation

	**AMP**	
	**A.**	**B.**
	B (*p*-value)	B (*p*-value)
Gender	0.25 (0.78)	0.55 (0.62)
Smoking status	−0.11 (0.90)	− 0.23 (0.81)
Δ FEV_1_ (L)	−0.97 (0.62)	− 0.74 (0.72)
Δ FEF_50_ (Ls^− 1^)	0.50 (0.54)	0.60 (0.48)
Δ R_20_ (kPa sL^−1^)	−2.00 (0.76)	− 1.01 (0.88)
Δ X_5_ (kPa sL^−1^)	−0.64 (0.67)	− 0.69 (0.65)
Δ LCI_5%_		0.22 (0.65)
	**Adenosine**	
	**A.**	**B.**
	B (*p*-value)	B (*p*-value)
Gender	−0.83 (0.37)	−0.81 (0.37)
Smoking status	0.32 (0.65)	0.45 (0.51)
Δ FEV_1_ (L)	1.50 (0.43)	0.98 (0.60)
Δ FEF_25–75_ (Ls^−1^)	−2.18 (0.04)	− 1.82 (0.09)
Δ R_20_ (kPa sL^− 1^)	−8.28 (0.11)	−6.53 (0.20)
Δ R_5_-R_20_ (kPa sL^− 1^)	2.61 (0.27)	3.20 (0.18)
Δ S_cond_		14.56 (0.16)

A. The models based on all subjects and B. the models incorporating multiple breath nitrogen washout (MBNW). Δ = change (post-pre); FEV_1_ = forced expiratory volume in the first second; FEF_50_ = forced expiratory flow at 50% of FVC; FEF_25–75_ = forced expiratory flow at 25 to 75% of FVC; R_20_ = resistance to 20 Hz; X_5_ = reactance to 5 Hz; R_5_-R_20_ = difference in resistance to 5 Hz and 20 Hz; LCI_5%_ = lung clearance index at 5%; S_cons_ = ventilation heterogeneity of the conducting airways

#### Spirometry-IOS-MBNW models

In the subgroup analysis incorporating MBNW data, ΔS_cond_ was added to the adenosine model (Table 2). The result shows that ΔBorg had the best association with ΔFEF_25–75_ (kPa sL^− 1^), yet not significant (*p* = 0.09). The model incorporating ΔS_cond_ had an improved R^2^ (R^2^ = 26.5%). The AMP model with MBNW incorporated ΔLCI_5%_ (Table 2), which shows no independent association to ΔBorg (R^2^ = 5.4%).

## Discussion

We found that dry powder adenosine and AMP evoke equal increases in dyspnea sensation, with a similar decrease in FEV_1_. However, during adenosine and AMP provocation, the increase in dyspnea sensation was differentially associated with large- and small airways dysfunction. The only independently association with dyspnea induced by dry powder adenosine was the decrease in FEF_25–75_, whereas dyspnea induced by AMP was not associated with changes in either large- or small airways dysfunction.

Our aim was to selectively target the small airways with dry powder adenosine. Therefore, we expected that dyspnea induced by dry powder adenosine would associate primarily with small airways parameters. Our findings were partly in line with this as we found the increase in Borg dyspnea score after inhalation of adenosine to associate with the decrease in FEF_25–75_, both in the univariate and multivariate analysis. However, the adenosine-induced change in other small airways parameters, such as R_5_-R_20_, S_cond_ and S_acin_, did not associate with ΔBorg. This was in contrast to our expectations, as these parameters are considered to be measures of the more peripheral small airways. A possible explanation could be that the measurements provide different information, yet there is no gold standard to determine which parameter is most accurate. Another possible explanation could be that the adenosine did not reach the more peripheral small airways even though it was designed to reach the small airways, consist of relatively small particles (MMAD of 2.6–2.9 μm) [20], and was inhaled with a low flow of 30 L/min [21]. Unfortunately we lack information on the exact deposition as radiolabeling for adenosine was not performed and our conclusions are thus based on assumed differential deposition.

With respect to AMP-induced dyspnea, multivariate analysis showed no large or small airways parameters that independently associated with ΔBorg. This may suggest that other factors than airway caliber or resistance play a role in the sensation of induced dyspnea. AMP acts on adenosine receptors which are located on various inflammatory cells including mast cells, eosinophils, and neutrophils, and their activation induces a cascade resulting in airway contraction [12]. Adenosine receptors are also found on afferent nerve endings [26]. It could be speculated that activation of afferent nerve endings plays a role in the dyspnea sensation after inhalation of AMP, independent of the presence of airway contraction. This activation may be direct or indirect through bronchial interstitial edema. In the context of direct activation, the findings of Burki et al. [27] are of interest. They administered intravenous adenosine to six asthmatic and six healthy subjects. Both groups reported a significant increase in dyspnea, with a higher intensity of the dyspnea in asthmatics. The FEV_1_, however, remained unchanged, indicating the absence of airway constriction. Based on these observations, they concluded that afferent nerve endings may be involved in the adenosine-induced sensation of dyspnea, which in asthmatics might be sensitized due to inflammation. In the context of indirect activation, interstitial edema may arise when the adenosine-induced inflammatory response induces alveolar-capillary leakage [28], which triggers the J-receptors to induce dyspnea sensation [29]. This, combined with the knowledge that afferent nerve endings are mainly seen in the upper and central airways [30], where we assume AMP primarily deposits, supports our speculation.

Although dry powder adenosine and AMP provocation may induce dyspnea through different processes, the degree of dyspnea after the final dose was not different. In addition, both tests were well tolerated and, apart from dyspnea, only led to minor cough in some subjects. This confirms previous findings in a small proof of concept study, that the relatively new adenosine provocation test is well tolerated [23].

We only included current and ex-smokers with asthma. It is therefore unclear whether these findings can be extrapolated to never-smoking asthmatics, as previous studies have shown a decreased dyspnea perception attributed to smoking, in asthmatics [31]. Never-smoking asthmatics may have had greater increases in dyspnea as a result of the provocations, but what this would have done to the association of dyspnea to large- and small airways parameters cannot be speculated.

## Conclusion

Our study shows that provocation with dry powder adenosine and AMP evoke similar levels of dyspnea. Dyspnea sensation evoked with dry powder adenosine shows small airways involvement independent of large airways involvement, while AMP evoked dyspnea associated with neither large- nor small airways dysfunction. This may indicate that dry powder adenosine and AMP evoke dyspnea via different processes.

## Additional file

Additional file 1: This document contains supplementary Tables S1 and S2, as referred to in the text. **Table S1** is The change (post - pre) in all pulmonary function parameters evoked by the provocation. **Table S2** shows Spearman’s univariate correlation of the change in Borg dyspnea score with gender, smoking status, and all pulmonary function parameters. (DOCX 27 kb)

## Abbreviations

AHR: Airway hyperresponsiveness
AMP: Adenosine 5′-monophosphate
AX: Reactance area
FEF_25_: Forced expiratory flow at 25% of FVC
FEF_25–75_: Forced expiratory flow between 25 and 75% of the expiration
FEF_50_: Forced expiratory flow at 50% of FVC
FEF_75_: Forced expiratory flow at 75% of FVC
FEV_1_: Forced expiratory volume in the first second
FRC: Functional residual capacity
FVC: Forced vital capacity
IOS: Impulse oscillometry
LCI: Lung clearance index
MMAD: Mass median aerodynamic diameter
PC_20_: Provocative concentration causing the FEV_1_ to drop ≥20%
PD_20_: Provocative dose causing the FEV_1_ to drop ≥20%
R_20_: Airway resistance to 20 Hz
R_5_: Airway resistance to 5 Hz
R_5_-R_20_: Difference between airway resistance to 5 Hz and 20 Hz
SABA: Short acting β_2_-antagonists
S_acin_: Ventilation heterogeneity of the acinar airways
S_cond_: Ventilation heterogeneity of the conductive airways
TLC: Total lung capacity
X_5_: Reactance to 5 Hz
Δ: Change; pre-provocation minus post-provocation
