# Supplementary material for: Associations of AMP and adenosine induced dyspnea sensation to large and small airways dysfunction in asthma
Source: BMC Pulm Med. 2019 Jan 28;19:23. doi: 10.1186/s12890-019-0783-0 (PMC6348600; doi:10.1186/s12890-019-0783-0)
Supplement: Supplementary file 1 — This document contains supplementary Tables S1 and S2, as referred to in the text. Table S1 is The change (post - pre) in all pulmonary function parameters evoked by the provocation. Table S2 shows Spearman’s univariate correlation of the change in Borg dyspnea score with gender, smoking status, and all pulmonary function parameters. (DOCX 27 kb) [file 12890_2019_783_MOESM1_ESM.docx]

## Supplementary Table S1: The change (Δ, post - pre) evoked by the provocation.

|  | **Adenosine** | **AMP** | **p- value** |
| --- | --- | --- | --- |
| **Δ Borg^#^** | 3.95 (2.07) | 3.77 (2.11) | 0.65^t^ |
| **Δ FEV_1_ (L)^#^** | -0.68 (0.30) | -0.62 (0.28) | 0.07^t^ |
| **Δ FEF_25_ (Ls^-1^)^#^** | -1.34 (-2.27; -0.84) | -1.18 (-1.78; -0.75) | 0.36^w^ |
| **Δ FEF_50_ (Ls^-1^)^@^** | -0.63 (-1.14; -0.40) | -0.65 (-1.01; -0.33) | 0.64^w^ |
| **Δ FEF_75_ (Ls^-1^)^@^** | -0.17 (-0.34; -0.07) | -0.15 (-0.35; -0.02) | 0.51^w^ |
| **Δ FEF_25-75_ (Ls^-1^)^@^** | -0.47 (-0.92; -0.29) | -0.47 (-0.84; -0.27) | 0.40^w^ |
| **Δ FVC (L)^#^** | -0.73 (0.44) | -0.66 (0.39) | 0.27^t^ |
| **Δ R_5_ (kPa sL^-1^)^#^** | 0.33 (0.23) | 0.35 (0.24) | 0.76^t^ |
| **Δ R_20_ (kPa sL^-1^)^#^** | 0.05 (0.08) | 0.07 (0.09) | 0.04^t^ |
| **Δ R_5_-R_20_ (kPa sL^-1^)^#^** | 0.27 (0.20) | 0.28 (0.22) | 0.81^t^ |
| **Δ AX (kPa L^-1^)^@^** | 2.97 (1.84; 5.15) | 3.31 (1.72; 5.83) | 0.50^w^ |
| **Δ X_5_ (kPa sL^-1^)^#^** | -0.32 (0.19) | -0.33 (0.26) | 0.64^t^ |
| **Δ F_res_ (s^-1^)^#^** | 10.03 (8.22) | 10.85 (6.92) | 0.63^t^ |
| **Δ LCI_2.5%_^@^*** | 3.42 (2.01; 4.58) | 2.83 (1.63; 5.45) | 0.03^w^ |
| **Δ LCI_5%_^@^*** | 1.53 (0.89; 2.32) | 1.46 (0.65; 2.71) | 0.07^w^ |
| **Δ S_cond_^#^*** | 0.02 (0.04) | 0.03 (0.03) | 0.58^t^ |
| **Δ S_acin_^@^*** | 0.12 (0.03; 0.27) | 0.05 (0.03; 0.12) | 0.01^w^ |

Data is presented as mean with standard deviation (#) or median with inter quartile range (@).* = multiple breath nitrogen washout (MBNW) was measured in 36 subjects. Comparison is either done with a paired t-test (t) or a Wilcoxon signed rank test (w). Δ = post provocation – pre provocation value; FEV_1_= forced expiratory volume in the first second; FVC = forced vital capacity; FEF_25_= forced expiratory flow at 25% of FVC; FEF_50_= forced expiratory flow at 50% of FVC; FEF_75_= forced expiratory flow at 75% of FVC; FEF_25-75_= forced expiratory flow at 25% to 75% of FVC; R_5_= resistance to 5Hz; R_20_= resistance to 20Hz; R_5_-R_20_= difference in resistance to 5Hz and 20Hz; AX= reactance area; X_5_= reactance to 5Hz; F_res_= resonance frequency; S_cons_= ventilation heterogeneity of the conducting airways; S_acin_= ventilation heterogeneity of the acinar airways.

## Supplementary Table S2: Spearman’s univariate correlation for the change in Borg (ΔBorg).

|  | Adenosine | AMP |
| --- | --- | --- |
| Gender | 0.08 (0.54) | -0.05 (0.72) |
| Smoking status | 0.15 (0.26) | 0.00 (0.99) |
| Δ FEV_1_ (L) | -0.17 (0.21) | -0.23 (0.10) |
| Δ FEF_25_ (Ls^1^) | -0.32 (0.01) | -0.21 (0.14) |
| Δ FEF_50_ (Ls^1^) | -0.25 (0.06) | -0.11 (0.42) |
| Δ FEF_75_ (Ls^1^) | -0.37 (<0.01) | 0.01 (0.92) |
| Δ FEF_25 75_ (L) | -0.34 (<0.01) | -0.05 (0.71) |
| Δ FVC (L) | -0.02 (0.87) | -0.02 (0.88) |
| Δ R_5_ (kPa sL^-1^) | 0.13 (0.33) | 0.21 (0.13) |
| Δ R_20_ (kPa sL^-1^) | -0.03 (0.83) | -0.04 (0.76) |
| Δ R_5_-R_20_ (kPa sL^-1^) | 0.20 (0.14) | 0.23 (0.10) |
| Δ AX (kPa sL^-1^) | 0.10 (0.47) | 0.28 (0.05) |
| Δ X_5_ (kPa sL^-1^) | -0.01 (0.93) | -0.30 (0.04) |
| Δ F_res_ (kPa sL^-1^) | 0.14 (0.31) | -0.00 (0.99) |
| Δ LCI_2.5%_* | 0.07 (0.71) | -0.00 (0.99) |
| Δ LCI_5%_* | 0.12 (0.54) | 0.12 (0.54) |
| Δ S_cond_* | 0.24 (0.16)  ) | -0.04 (0.84) |
| Δ S_acin_* | -0.06 (0.76) | -0.07 (0.69) |

Δ = post provocation – pre provocation value; FEV_1_= forced expiratory volume in the first second; FVC = forced vital capacity; FEF_25_= forced expiratory flow at 25% of FVC; FEF_50_= forced expiratory flow at 50% of FVC; FEF_75_= forced expiratory flow at 75% of FVC; FEF_25-75_= forced expiratory flow at 25% to 75% of FVC; R_5_= resistance to 5Hz; R_20_= resistance to 20Hz; R_5_-R_20_= difference in resistance to 5Hz and 20Hz; AX= reactance area; X_5_= reactance to 5Hz; F_res_= resonance frequency; S_cons_= ventilation heterogeneity of the conducting airways; S_acin_= ventilation heterogeneity of the acinar airways. * = multiple breath nitrogen washout (MBNW) was measured in 36 subjects.
